# An mHealth Intervention to Improve Medication Adherence and Health Outcomes Among Patients With Coronary Heart Disease: Randomized Controlled Trial

**DOI:** 10.2196/27202

**Published:** 2022-03-09

**Authors:** Zhao Ni, Bei Wu, Qing Yang, Lijing L Yan, Changqing Liu, Ryan J Shaw

**Affiliations:** 1 School of Medicine Yale University New Haven, CT United States; 2 Rory Meyers College of Nursing New York University New York, NY United States; 3 School of Nursing Duke University Durham, NC United States; 4 Global Health Research Center Duke Kunshan University Kunshan China; 5 West China Hospital Sichuan University Chengdu China

**Keywords:** mHealth, medication adherence, coronary disease, blood pressure, China, randomized controlled trial

## Abstract

**Background:**

The treatment of many chronic illnesses involves long-term pharmaceutical therapy, but it is an ongoing challenge to find effective ways to improve medication adherence to promote good health outcomes. Cardioprotective medications can prevent the enlargement of harmful clots, cardiovascular symptoms, and poor therapeutic outcomes, such as uncontrolled high blood pressure and hyperlipidemia, for patients with coronary heart disease. Poor adherence to cardioprotective medications, however, has been reported as a global health concern among patients with coronary heart disease, and it is particularly a concern in China.

**Objective:**

This study aimed to evaluate the efficacy of a mobile health (mHealth) intervention using 2 mobile apps to improve medication adherence and health outcomes.

**Methods:**

A randomized, placebo-controlled, 2-arm parallel study was conducted in a major university-affiliated medical center located in Chengdu, China. Participants were recruited by flyers and health care provider referrals. Each participant was observed for 90 days, including a 60-day period of mHealth intervention and a 30-day period of nonintervention follow-up. The study coordinator used WeChat and Message Express to send educational materials and reminders to take medication, respectively. Participants used WeChat to receive both the educational materials and reminders. Participants in the control group only received educational materials. This study received ethics approval from the Duke Health Institutional Review Board (Pro00073395) on May 5, 2018, and was approved by West China Hospital (20170331180037). Recruitment began on May 20, 2018. The pilot phase of this study was registered on June 8, 2016, and the current, larger-scale study was retrospectively registered on January 11, 2021 (ClinicalTrials.gov).

**Results:**

We recruited 230 patients with coronary heart disease. Of these patients, 196 completed the baseline survey and received the intervention. The majority of participants were married (181/196, 92.4%), male (157/196, 80.1%), and lived in urban China (161/196, 82.1%). Participants’ average age was 61 years, and half were retired (103/191, 53.9%). More than half the participants (121/196, 61.7%) were prescribed at least 5 medications. The mean decrease in medication nonadherence score was statistically significant at both 60 days (t_179_=2.04, *P*=.04) and 90 days (t_155_=3.48, *P*<.001). Systolic blood pressure and diastolic blood pressure decreased in the experimental group but increased in the control group. The mean decrease in diastolic blood pressure was statistically significant at both 60 days (t_160_=2.07, *P*=.04) and 90 days (t_164_=2.21, *P*=.03). The mean decrease in systolic blood pressure was significantly different in the groups at 90 days (t_165_=3.12, *P*=.002).

**Conclusions:**

The proposed mHealth intervention can improve medication adherence and health outcomes, including systolic blood pressure and diastolic blood pressure.

**Trial Registration:**

ClinicalTrials.gov NCT02793830; https://clinicaltrials.gov/ct2/show/NCT02793830 and ClinicalTrials.gov NCT04703439; https://clinicaltrials.gov/ct2/show/NCT04703439

## Introduction

Coronary heart disease (CHD) is a heart and blood vessel disease related to atherosclerosis in which plaque builds up in the walls of the coronary arteries, narrowing the arteries and restricting the flow of blood [1]. CHD is the world’s leading cause of death [2], accounting for over one-third of all deaths in individuals over the age of 35 [3]. Approximately 4 out of 5 global deaths caused by CHD occur in low- and middle-income countries [4], and the burden of CHD is anticipated to increase in these countries [5]. By the year 2030, CHD is projected to cause more than 9 million deaths yearly [6]. China is the world’s largest low- or middle-income country [7,8], and in China, CHD is the second leading cause of death, accounting for over 1.5 million deaths per year [9,10]. CHD has been an enormous economic burden on Chinese society in terms of health care costs and loss of productivity, and the mortality rate of CHD is increasing [11,12]. The administration of cardioprotective medications is a key treatment modality for CHD and a preventive measure against cardiovascular events [13,14]. Cardioprotective medications, including antiplatelet drugs, beta-blockers, calcium channel blockers, statins, and angiotensin-converting enzyme inhibitors can reduce the mortality rate of CHD [15-17].

In China, however, poor adherence to cardioprotective medications has been reported as a public health concern [18], contributing to increased health care costs and a high mortality rate for cardiovascular diseases [14,19-21]. For example, studies in China have shown that 18.2% to 38% of patients with CHD did not adhere to their statin medications [22,23], and only 49% consistently used beta-blockers after hospital discharge [16,21]. The vast majority of highly qualified health care providers in China practice in large urban hospitals; for serious illness, such as CHD, patients prefer to utilize large hospitals rather than local primary health care clinics [24,25]. Under this treatment-focused health care utilization model, many patients with CHD in China receive prescriptions and medication-related education without a primary care clinician to monitor their medication-taking behavior [20]. Consequently, after being discharged from the hospital, patients often do not receive proper follow-up care or information regarding medication-taking behavior [19,23]; therefore, it is important to develop innovative interventions to address this issue. Mobile health (mHealth) is defined as the use of portable electronic devices with software applications to provide health care services and manage patient information [26-28]. China has 1.3 billion mobile phone users [29]; mobile technology is booming, with 97% of netizens using mobile phones to access the internet [30]. Pilot studies conducted in China have shown that it is feasible and acceptable to implement an mHealth intervention to improve medication adherence among patients with CHD [19].

After these pilot studies, mobile phone–based mHealth interventions have been conducted in the field of cardiovascular medicine in China [31-34], but none of them has been specifically aimed at improving medication adherence among patients with CHD, nor has any of them tested mHealth interventions that integrate 2 mobile apps. For example, in Shanghai, Dorje et al [31] conducted an mHealth intervention using social media to send participants educational materials to promote cardiac rehabilitation and secondary prevention, but the educational materials were not specifically about medication adherence. Also, reminders to take medication were not a primary intervention in this study; only participants whose blood pressure was outside the target level received reminders. In Guangzhou, Li et al [34] evaluated an mHealth intervention that aimed to improve blood pressure and self-management behavior in people with hypertension, but this intervention was neither specifically designed to improve medication adherence nor focused on patients with CHD. These studies indicate that although using mHealth interventions to improve medication adherence is a promising approach, more evidence is needed to examine the effect of mHealth intervention on improving medication adherence and clinical outcomes among patients with CHD [35]. The aim of this paper was to assess if our mobile phone–based mHealth intervention could improve medication adherence and relevant health outcomes (ie, systolic blood pressure, diastolic blood pressure, and heart rate) among patients with CHD in comparison to a control group that received general educational materials over a period of 60 days. The pilot phase of this study was registered in ClinicalTrials.gov (NCT02793830) on June 8, 2016, and the current, larger-scale study was retrospectively registered (NCT04703439) on January 11, 2021. This was because we wanted to make the study more accessible to the public and also because we believed that this would better reflect the continuousness of our mHealth intervention, from the initial evaluation of its feasibility and acceptability (ie, the pilot phase) to the later evaluation of its efficacy (ie, the larger-scale phase).

## Methods

### mHealth Technologies

This study used 2 mobile apps: WeChat (Tencent Inc) and Message Express (Bluemobile.zt). WeChat is the most widely used messaging app in China [36]. Message Express (also known as “Xiao Xi Su Di” in Chinese) is a message saving and delivery app that can send personalized messages to a WeChat account. It can be integrated with WeChat to deliver reminders to take medication from a message library. After saving a library of reminders to Message Express, a clinician can easily choose personalized reminders in Message Express and send them to a participant’s WeChat. BB Reminder is a third-party app with similar functions to Message Express that we used in the pilot study, but it became unavailable after April 2018. Thus, in this study, our study coordinator used WeChat and Message Express to send educational materials and reminders to take medication, respectively. Participants used WeChat to receive both educational materials and reminders. At baseline, we assessed the mobile phone literacy of participants by recording the length of time they had been using WeChat, their frequency of WeChat use, and their educational level. To protect participants’ data and privacy, we followed the patient privacy policies of West China Hospital and Duke University and used an encrypted cellphone to send messages. Participants’ data were stored in a secure database (REDCap, Vanderbilt University). No data were stored in WeChat or Message Express.

### Study Design and Participants

This unblinded, 2-arm, parallel randomized controlled trial was conducted between May and December 2018 at the Cardiology Department of West China Hospital, located in Chengdu. West China Hospital is a major university-affiliated hospital that serves more than 10,000 outpatients a day [37]. A total of 230 participants were recruited for this study using flyers and health care provider referrals [19]. The sample size was determined by a statistical power analysis (Gpower 3.1), with an α of 0.05, power of 0.80, and effect size of 0.35. The effect size was calculated using Cohen’s criteria based on data from phase II of the pilot study [38,39]. Written consent was obtained from participants, and they were randomly assigned to the experimental group using stratified randomization by gender with a permuted block size of 4. The random allocation sequence was generated using SAS software version 9.4 by the first author (ZN), who also enrolled participants and assigned participants to interventions. Each participant was observed for 90 days, including a 60-day period of mHealth intervention and a 30-day period of nonintervention follow-up. The mHealth intervention was developed and tested in a pilot study with 2 phases, in 2016 and 2017 [19]. In the pilot study, we found it was feasible to conduct an mHealth intervention by integrating 2 apps to remind patients with CHD to take medications. The apps that we chose to use in this study were based on the results of the pilot study. Participants were included in this study if they satisfied the following criteria [19]: (1) they had a medical diagnosis of CHD; (2) they were aged 18 years or older; (3) they had an antihypertensive medication regimen that would last for 90 days or more after enrollment; (4) they were able to read messages through a mobile phone; (5) they had a mobile phone that could receive messages from WeChat and reminders from Message Express; (6) they were capable of giving consent; and (7) they had an electronic blood pressure cuff to check blood pressure and heart rate.

### Ethics Approval

This study received ethical approval from the Duke Health Institutional Review Board (Pro00073395) and West China Hospital (20170331180037).

### Interventions

**Figure 1:**
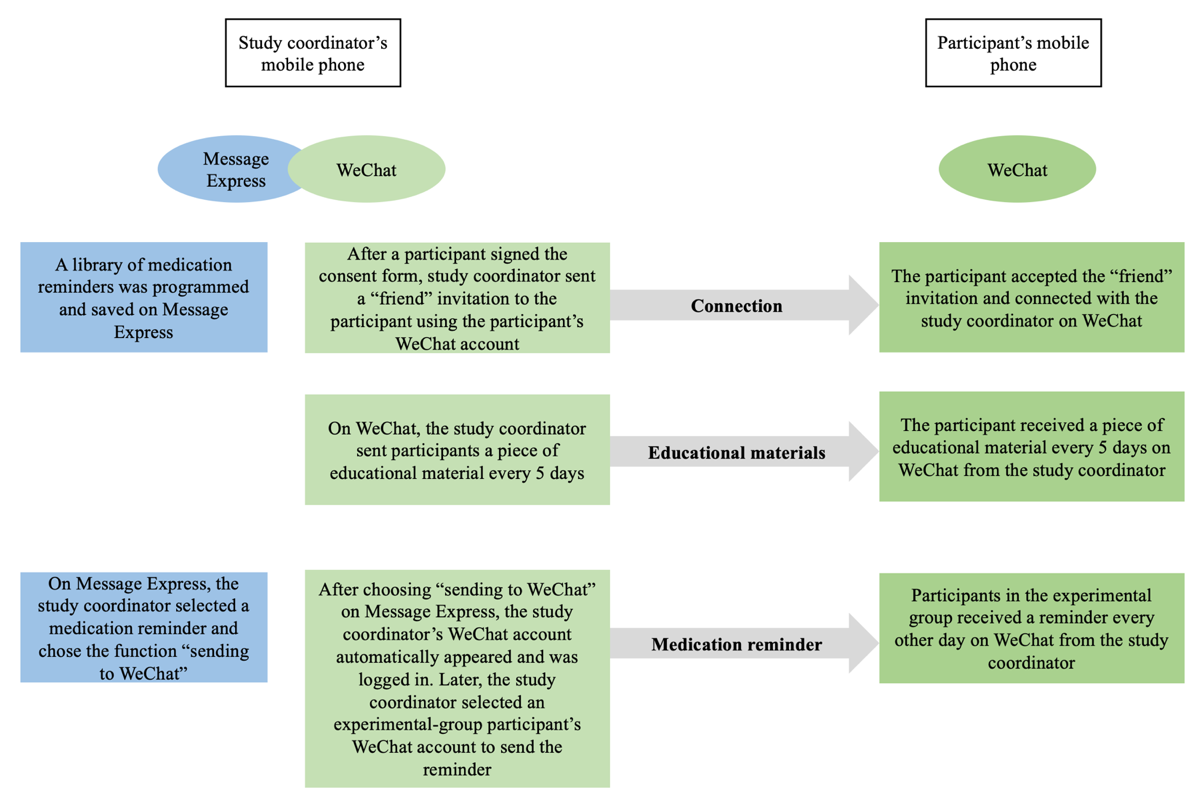
Brief description of the 2 apps.

The interventions were refined based on the pilot study [19]. Once a participant completed the baseline questionnaire, they received the allocated intervention. Participants in the experimental group received reminders to take medication and educational materials from Message Express and WeChat, respectively (Figure 1). Participants in the control group only received educational materials from WeChat. The educational materials were sent every 5 days at a random time between 8 AM and 9 AM. The educational materials sent to the 2 groups were different. Materials sent to the experimental group were specifically related to CHD and medication adherence and included information on the function of cardioprotective medications, potential negative consequences of not taking these medications daily, and some common reasons why people with CHD fail to take them. Materials sent to the control group contained general medical information that was not specifically related to CHD or medication adherence. For example, some of the materials were related to population aging in China and age-related health problems, such as hearing loss. All educational materials were retrieved from the website of the World Health Organization [40,41] and were screened by a cardiologist and a nurse to ensure their accuracy before being sent to participants. In addition to educational materials, participants in the experimental group received reminders to take medication every morning at a random time between 7 AM and 8 AM. (The reminders included phrases such as “Please remember to take today’s medications” and “It is time to take today’s medications. Do not stop taking your medications without consulting a cardiologist.”) The time participants received the reminders was selected based on feedback that we collected in the pilot study. The educational materials and reminders were sent through Message Express on an encrypted external device (an iPhone 5 borrowed from Duke University that was used specifically for this study). The intervention lasted for 60 days for each participant. After the intervention, participants were observed for 30 days.

### Data Collection and Variables

Participants’ demographic characteristics were recorded, including age, gender, ethnicity, weight, height, marital status, job status, education, medical insurance status, living area, number of prescribed medications, and family income. A REDCap survey hyperlink was sent to each participant to collect their demographic characteristics and track their health outcome variables at 7 time points: enrollment (ie, baseline); 15, 30, 45, and 60 days after enrollment (ie, at the end of the intervention); and 75 and 90 days after enrollment (ie, at the end of follow-up). Health outcome variables included medication nonadherence score, heart rate (HR), systolic blood pressure (SBP), and diastolic blood pressure (DBP). The primary outcome (ie, medication nonadherence score) was measured using a validated 3-item, 5-point Likert scale, the Voils Extent Scale [42]. To calculate the total nonadherence score, the responses to the relevant 3 items of the survey were added up. A lower score indicated better medication adherence. If a participant did not complete a survey, the study coordinator sent them a WeChat message as a reminder. If there was no reply after 2 such attempts, the study coordinator called the participant. If there was still no answer, the participant was treated as lost to follow-up. Participants who deleted the study coordinator’s WeChat account were treated as having withdrawn from the study.

### Data Analysis

All analyses were performed using SAS 9.4. Categorical variables such as gender, ethnicity, marital status, job status, medical insurance, and living area were reported as frequency (n) and percentage (%). Numerical variables including age, weight, height, and number of prescribed medications were reported as mean (SD). The chi-square test and independent *t* tests were used to determine between-group differences at baseline. The Fisher exact test and Wilcoxon 2-sample test were used as alternatives when a chi-square test was not applicable due to small expected values in some cells or when a 2-sample *t* test assumption was not met. All statistical tests were nondirectional (2-tailed) and the significance level was set at *P*=.05. If a difference in a baseline characteristic was significant, the baseline variable was included as a covariate in the modeling stage.

The mean (SD) for all outcome variables (ie, medication nonadherence, HR, SBP, and DBP) in each group was computed and graphed to show trends at the 7 time points described above. Differences in outcomes between the experimental and control groups at the critical time points (ie, baseline, 60 days, and 90 days) were determined using the *t* test. Both the control and experimental groups contained individuals with extremely low or high blood pressure and HR, and calculating the mean of these abnormal values was likely to produce values that were misleadingly normal. Therefore, we dichotomized SBP, DBP, and HR values into binary variables: normal and abnormal. We defined normal blood pressure according to the 2010 Chinese Hypertension Guidelines [43], which define normal SBP and DBP as less than 140 mmHg and less than 90 mmHg, respectively, and define normal HR as between 60 bpm and 100 bpm. The proportional rates of normal HR, SBP, and DBP in the subjects were graphed to show trends. To test between-group differences in the trajectory of change in all outcomes after adjusting for relevant covariates, a mixed-effects model was used for continuous outcomes and a generalized mixed-effects model with a logit link was used for the dichotomized binary outcomes. Considering the correlations between repeated longitudinal measurements of the same patient, a random intercept, a random slope, and an unstructured covariate matrix were used. The fixed effects included in the 2 models were the intervention group (ie, experimental versus control), time, the group-by-time interaction, and baseline variables that were significantly different in the experimental and control groups or covariates that could clinically impact HR and blood pressure. We also accommodated a quadratic time trend by including a time-by-time fixed effect and testing for it. If the quadratic effect was not significant, we removed it from the mixed-effects model.

## Results

### Baseline Characteristics

**Figure 2:**
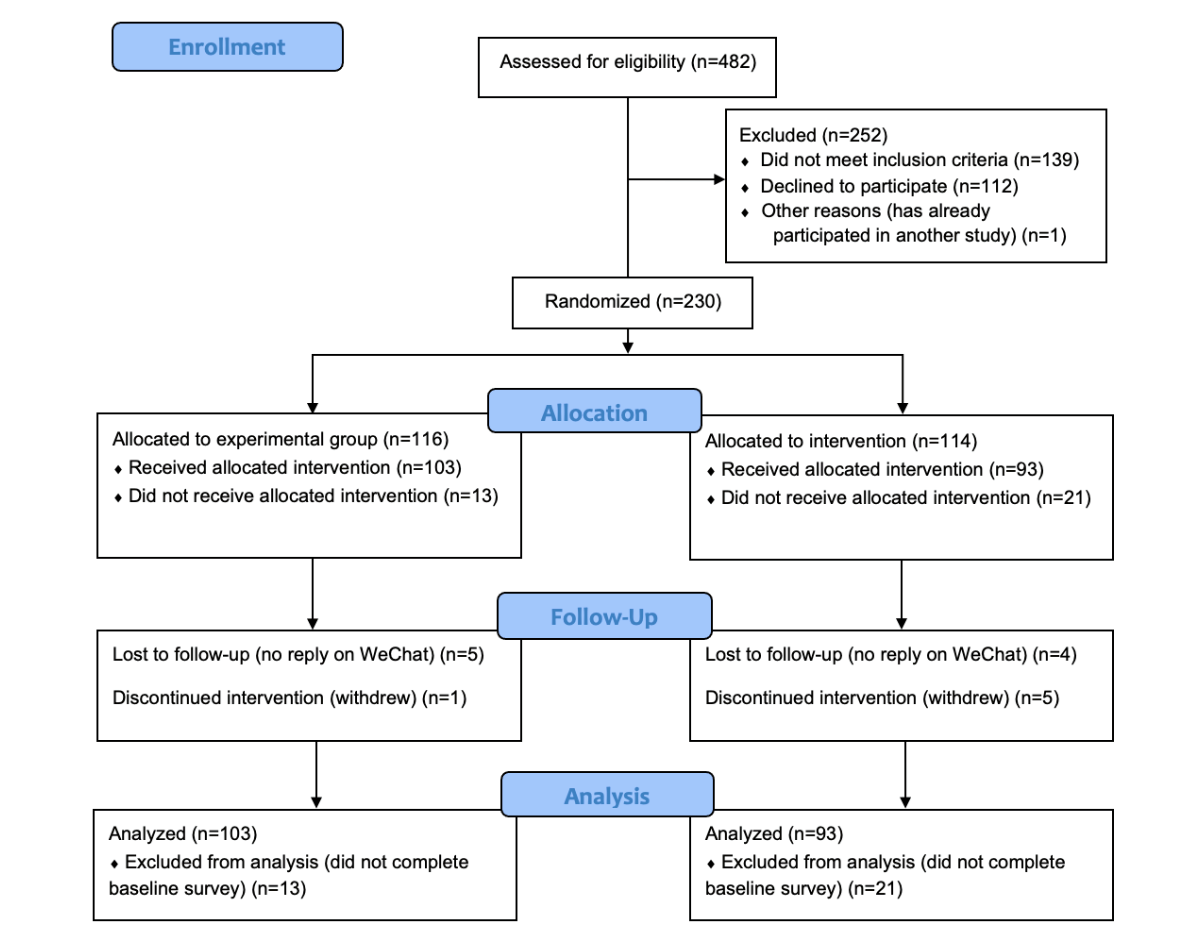
CONSORT (Consolidated Standards of Reporting Trials) flow diagram.

In this study, we recruited 230 participants and randomly assigned 116 to the experimental group and 114 to the control group (see Figure 2 for the CONSORT [Consolidated Standards of Reporting Trials] diagram [44]). Of the 230 participants, 34 participants did not complete the baseline questionnaire and thus did not receive the intervention. We collected baseline data from the 196 participants who received the intervention; of these, 6 participants later dropped out of the study and 9 were lost during the follow-up period. There was no statistically significant difference (*P*>.05) in any baseline characteristic between the 2 groups (Table 1). The majority of the participants were married (181/196, 92.4%), male (157/196, 80.1%), Han Chinese (184/196, 93.9%), and living in urban China (161/196, 82.1%). Participants’ average age was 61 years, and half were retired (103/191, 53.9%). More than half the participants (121/196, 61.7%) were prescribed at least 5 medications. Half the participants had an annual family income less than CNY ¥54,000 (US $7950). The majority of participants (186/192, 96.9%) had medical insurance; nearly 28% (41/147) of participants had their medications fully covered, 60% (89/147) were partially covered, and 11.6% (17/147) of participants did not have insurance coverage for their medication. Most participants (109/196, 88%) had used WeChat for more than a year and were using WeChat every day (152/188, 80.9%) before participating in the study; only 5.8% (11/188) had never used WeChat before the study. There was no difference between the experimental and control groups in mobile phone literacy.

**Table 1:** Baseline characteristics of participants at enrollment.

Variable		All participants (N=196)	Experimental group (n=103)	Control group (n=93)	*P* value
**Gender, n (%)**					.86
	Male	157 (80.1)	83 (80.6)	74 (79.6)	
	Female	39 (19.9)	20 (19.4)	19 (20.4)	
Age (years), mean (SD)		61 (11)	61 (11)	62 (11)	.48
Height (cm), mean (SD)		164.8 (8.0)	165.5 (8.0)	164.1 (8.0)	.23
Weight (kg), mean (SD)		67.6 (11.3)	68.2 (11.9)	66.9 (10.5)	.45
**Ethnicity, n (%)**					.98^a^
	Han	184 (93.9)	96 (93.2)	88 (94.6)	
	Zang	6 (3.1)	3 (2.9)	3 (3.2)	
	Yi	1 (0.5)	1 (1.0)	0 (0)	
	Hui	1 (0.5)	0 (0)	1 (1.1)	
	Mongolian	1 (0.5)	1 (1.0)	0 (0)	
	Other ethnic minorities	3 (1.5)	2 (1.9)	1 (1.1)	
**Marital status, n (%)**					.25
	Married	181 (92.4)	93 (90.3)	88 (94.6)	
	Widowed, separated, divorced, or single	15 (7.6)	10 (9.7)	5 (5.4)	
**Job status, n (%)**					.31^a^
	Employed	65 (34.0)	39 (39.0)	26 (28.6)	
	Unemployed	4 (2.1)	3 (3.0)	1 (1.1)	
	Farmer	19 (10.0)	8 (8.0)	11 (12.1)	
	Retired	103 (53.9)	50 (50.0)	53 (58.2)	
**Education, n (%)**					.36
	Primary school or lower	31 (16.1)	16 (15.8)	15 (16.3)	
	Middle school	43 (22.3)	27 (26.7)	16 (17.4)	
	High school	47 (24.3)	26 (25.7)	21 (22.8)	
	Noncollege postsecondary	28 (14.5)	11 (10.9)	17 (18.5)	
	College or above	44 (22.8)	21 (20.8)	23 (25.0)	
**Number of prescribed medications, n (%)**					.82^a^
	<5	75 (38.3)	41 (39.8)	34 (36.6)	
	5-9	116 (59.2)	59 (57.3)	57 (61.3)	
	≥10	5 (2.5)	3 (2.9)	2 (2.1)	
Covered by medical insurance		186 (96.9)	97 (96.0)	89 (97.8)	.69^a^
**Medication coverage as part of medical insurance, n (%)**					.90^b^
	Complete coverage	41 (27.9)	22 (30.1)	19 (25.7)	
	Some coverage	89 (60.5)	40 (54.8)	49 (66.2)	
	No coverage	17 (11.6)	11 (15.1)	6 (8.1)	
**Place of residence, n (%)**					.82
	Urban	161 (82.1)	84 (81.6)	77 (82.8)	
	Rural	35 (17.9)	19 (18.4)	16 (17.2)	
**Living arrangements, n (%)**					.36
	Living alone	14 (7.1)	9 (8.7)	5 (5.4)	
	Living with family or relatives	182 (92.9)	94 (91.3)	88 (94.6)	
**Length of using WeChat, n (%)**					.78
	<1 year	24 (12.0)	11 (10.7)	13 (14.0)	
	1-4 years	109 (56.0)	58 (56.3)	51 (54.8)	
	≥5 years	63 (32.0)	34 (33.0)	29 (31.2)	
**Frequency of WeChat use before participating in the study, n (%)**					.44
	Daily	152 (80.9)	83 (83.8)	69 (77.5)	
	Occasionally	25 (13.3)	12 (12.1)	13 (14.6)	
	Never	11 (5.8)	4 (4.0)	7 (7.9)	
**Annual family income (CNY¥), n (%)**					.12^b^
	<¥54,000	81 (52.3)	48 (59.3)	33 (44.6)	
	¥54,001-¥90,000	34 (21.9)	13 (16.1)	21 (28.4)	
	¥90,001-¥120,000	18 (11.6)	11 (13.6)	7 (9.5)	
	>¥120,000	22 (14.2)	9 (11.1)	13 (17.6)	
**General health status, n (%)**					.89
	Good	70 (35.7)	37 (35.9)	33 (35.5)	
	Fair	101 (51.5)	54 (52.4)	47 (50.5)	
	Bad	25 (12.8)	12 (11.7)	13 (14.0)	

^a^The Fisher exact test was used due to small values in cells.

^b^The Mann-Whitney *U* test was used due to missing data: 25% of participants did not know whether their prescribed medications would be covered by their medical insurance at the time of discharge; and 21% of participants refused to disclose their family income.

### Medication Nonadherence and Health Outcomes

**Figure 3:**
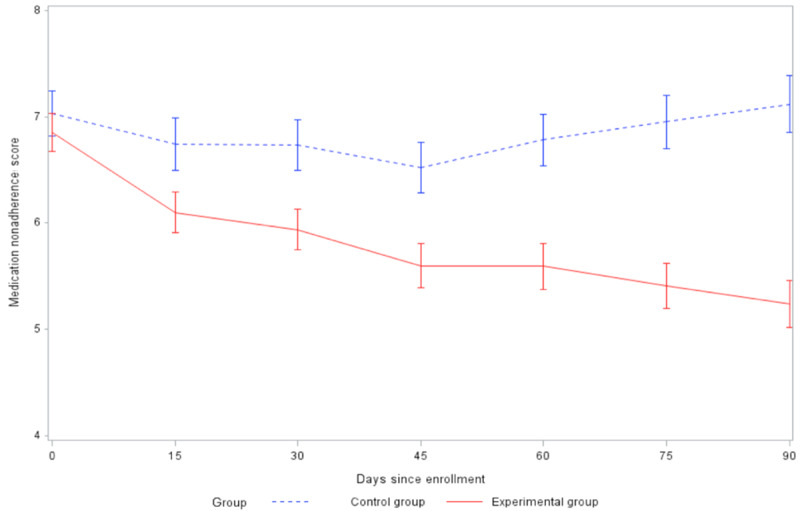
Comparison of changes in medication nonadherence score between groups (SE 2).

The medication nonadherence score (Figure 3) consistently decreased in the experimental group. The nonadherence score decreased in the control group during the first 45 days, but then increased and approached the baseline level at 90 days (the score at 90 days was 0.08 less than baseline). The mean decrease in medication nonadherence score in the experimental group was greater than the mean decrease in the control group at 60 days (t_179_=2.04, *P*=.04) and 90 days (t_155_=3.48, *P*<.001) (Table 2).

**Figure 4:**
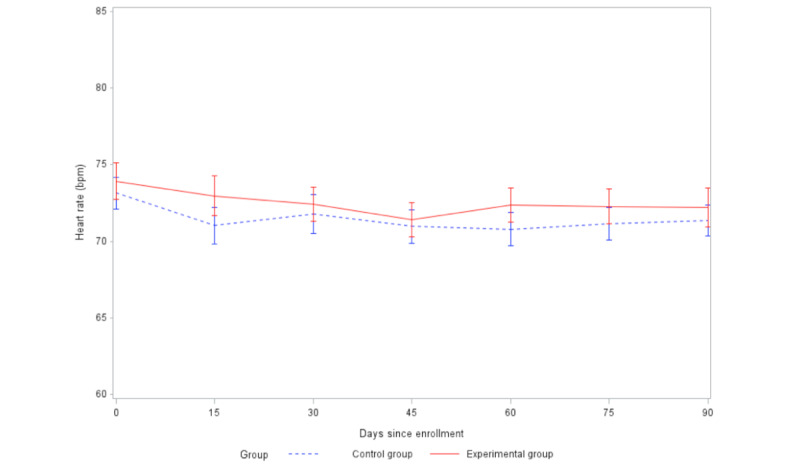
Comparison of changes in heart rate between groups (SE 2).

**Figure 5:**
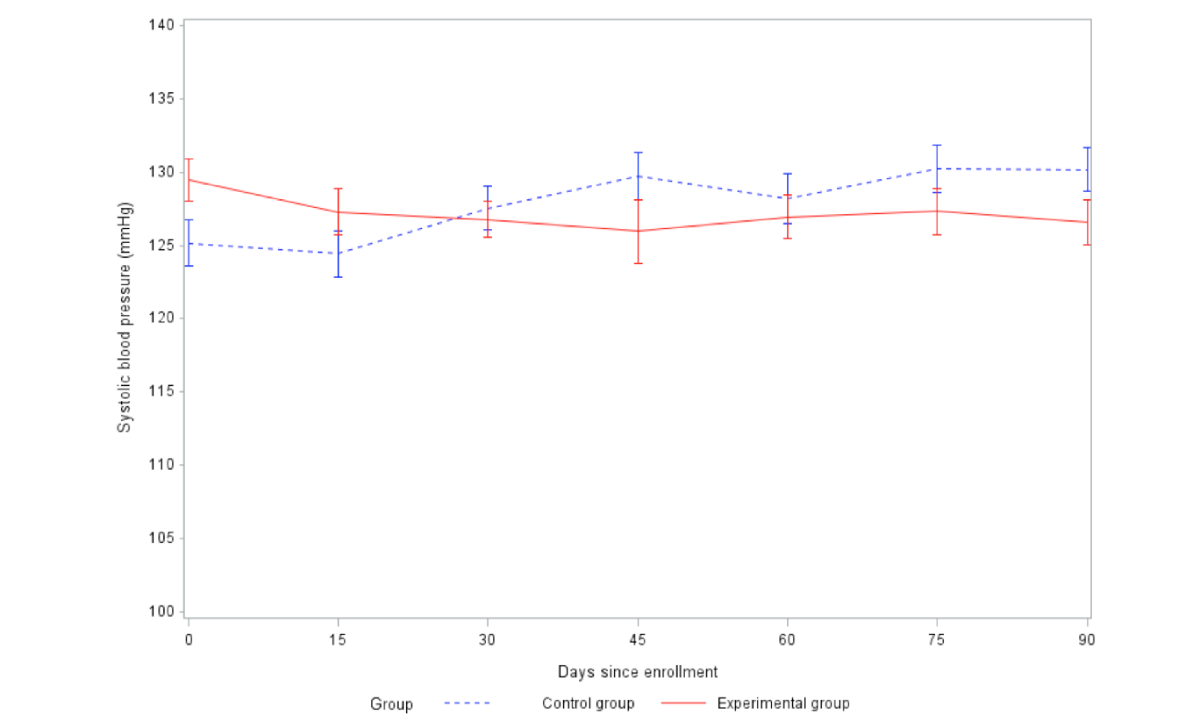
Comparison of changes in systolic blood pressure between groups (SE 2).

**Figure 6:**
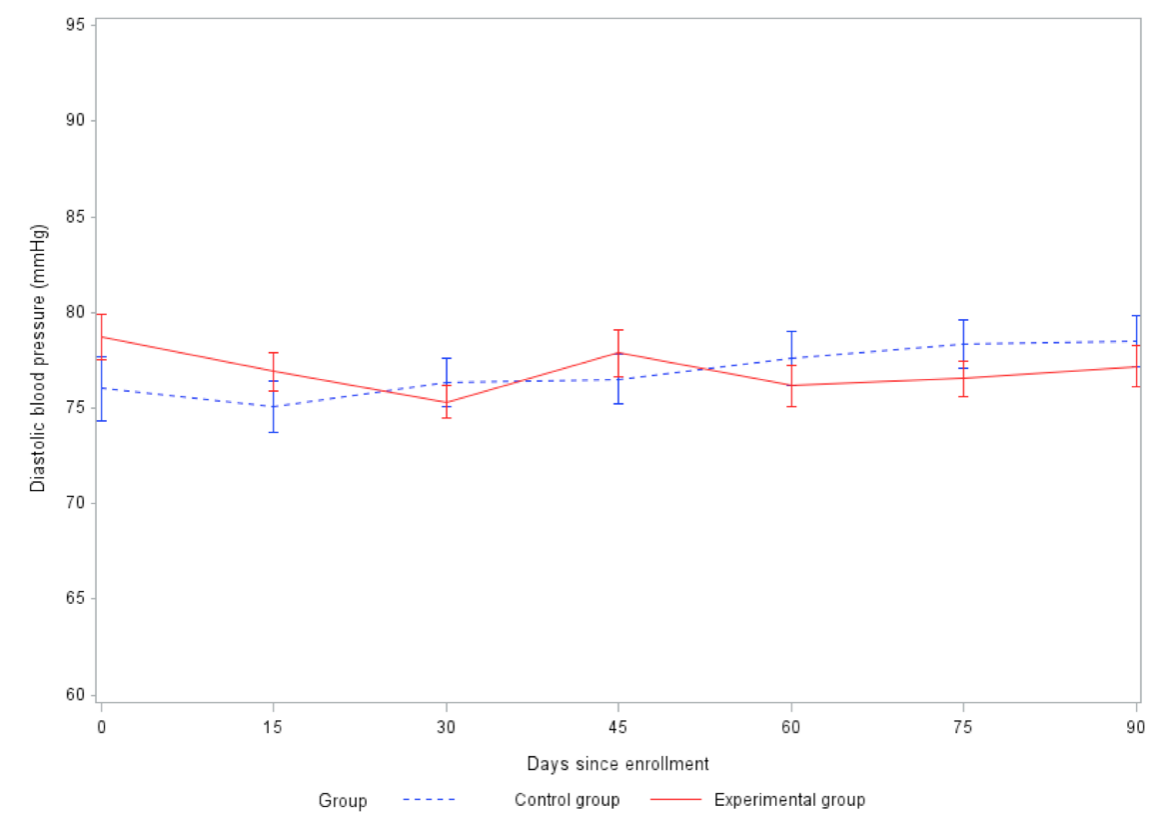
Comparison of changes in diastolic blood pressure between groups (SE 2).

HR decreased (Figure 4) at 60 days and 90 days in both groups compared to baseline, but the difference in the decrease between the 2 groups was not statistically significant at either 60 days (t_148_=–0.28, *P*=.78) or 90 days (t_145_=0.32, *P*=.75). SBP (Figure 5) and DBP (Figure 6) decreased at 60 days and 90 days in the experimental group but increased in the control group. The difference in the change in DBP between the intervention and control groups was statistically significant at both 60 days (t_160_=2.07, *P*=.04) and 90 days (t_164_=2.21, *P*=.03). The difference in the change in SBP between the 2 groups was statistically significant at 90 days (t_165_=3.12, *P*=.002), but not significant at 60 days (t_161_=1.92, *P*=.06).

The proportional rate of participants with normal SBP (Multimedia Appendix 1) and DBP (Multimedia Appendix 2) increased in both groups at 60 days and 90 days compared to baseline, but the difference between the 2 groups at these times was not statistically significant (Multimedia Appendix 3). Unlike SBP and DBP, the proportional rate of participants with normal HR (Multimedia Appendix 4) decreased in both the experimental and control groups, but the difference between the 2 groups was not statistically significant at either 60 days (*P*=.37) or 90 days (*P*=.41).

Although no baseline characteristics were significantly different between the experimental group and the control group, HR and blood pressure can be influenced by body weight [45,46], gender [47,48], and age [49]; therefore, they were included as covariates in the modeling stage. After controlling for the effects of baseline body weight, gender, age, educational level, group, time, and the group-by-time interaction in the mixed-effects model, the difference in the rate of change of medication nonadherence between the 2 groups was statistically significant (*β*=−.02, *P*<.001) (Table 3).

The difference in the rate of change of SBP (*β*=–.08, *P*<.001) and DBP (*β*=−.05, *P*=.004) between the 2 groups was also statistically significant. However, the difference in the rate of change in HR between the 2 groups was not statistically significant (*β*=–.01, *P*=.74). In the generalized mixed effect model with a logit link for dichotomized binary outcomes, we found that the difference in the change of the proportional rate of participants with a normal SBP between the 2 groups was statistically significant (*β*=.01, *P*=.02), but that the difference between the 2 groups was not statistically significant for either the proportional rate of participants with normal DBP (*β*=.006, *P*=.32) or normal HR (*β*=.003, *P*=.60) (Multimedia Appendix 5).

**Table 2:** Comparison of baseline and unadjusted changes in medication nonadherence and health outcomes between the 2 arms.

		Experimental group (n=103), mean (SD)	Control group (n=93), mean (SD)	*t* test (*df*)	*P* value
**Medication nonadherence score**					
	Baseline	6.85 (1.85)	7.03 (2.05)	0.64 (194)	.52
	Day 60	–1.21 (2.59)	–0.42 (2.63)	2.04 (179)	.04
	Day 90	–1.58 (2.49)	–0.08 (3.15)	3.48 (155)	<.001
**Heart rate (bpm)**					
	Baseline	73.93 (12.03)	73.16 (9.76)	–0.49 (183)	.63
	Day 60	–1.46 (12.68)	–1.95 (9.03)	–0.28 (148)	.78
	Day 90	–1.88 (12.88)	–1.32 (8.98)	0.32 (145)	.75
**Systolic blood pressure (mmHg)**					
	Baseline	129.5 (14.37)	125.2 (15.10)	–2.04 (188)	.04
	Day 60	–2.14 (16.20)	2.72 (16.07)	1.92 (161)	.06
	Day 90	–2.87 (15.10)	4.38 (14.89)	3.12 (165)	.002
**Diastolic blood pressure (mmHg)**					
	Baseline	78.71 (11.73)	76.03 (15.98)	–1.30 (162)	.20
	Day 60	–2.46 (12.49)	1.87 (14.06)	2.07 (160)	.04
	Day 90	–1.57 (12.21)	2.92 (13.99)	2.21 (164)	.03

**Table 3:** Results of a mixed-effects model with a random intercept and slope to measure between-group differences in the trajectory of changes in outcomes.

Item	Medication nonadherence		Diastolic blood pressure (mmHg)		Systolic blood pressure (mmHg)		Heart rate (bpm)	
	Parameter estimation	*P* value	Parameter estimation	*P* value	Parameter estimation	*P* value	Parameter estimation	*P* value
Intercept	5.11	<.001	84.86	<.001	93.48	<.001	56.66	<.001
Group (reference: control)	–0.23	.37	2.05	.19	3.39	.06	0.68	.63
Gender (reference: male)	0.29	.33	–2.50	.15	0.23	.92	2.00	.26
Age	0.002	.85	–0.22	<.001	0.26	.001	0.001	.99
Education	0.11	.17	0.17	.72	0.41	.50	–0.21	.66
Weight	0.013	.27	0.11	.12	0.21	.02	0.21	.003
Time (days)	0.001	.74	0.04	.14	0.06	.09	–0.01	.33
Group × time	–0.02	<.001	–0.05	.004	–0.08	<.001	–0.01	.74

## Discussion

### Principal Findings

The aim of this study was to assess if a mobile phone–based mHealth intervention could improve medication adherence and relevant health outcomes (eg, blood pressure and HR) among patients with CHD in comparison to a control group that received general educational materials over a period of 2 months. We found that our mHealth intervention increased medication adherence and had a lasting effect in improving medication adherence among patients with CHD, even though no intervention was given after 60 days. We also found that our mHealth intervention improved health outcomes by lowering SBP and DBP, and that this effect continued for 30 days after the intervention. Unlike SBP and DBP, our mHealth intervention did not significantly lower HR, although mean HR consistently remained within the normal range. After dichotomizing SBP, DBP, and HR into binary variables, we found that there was no significant difference between the proportional rate of participants with normal SBP, DBP, and HR at baseline, 60 days, or 90 days between the intervention and control groups.

A mixed-effects model for continuous outcomes showed that differences in the rate of change of medication nonadherence, SBP, and DBP were statistically significant between the 2 groups after controlling for the effects of group, time, the group-by-time interaction, and some baseline variables that can influence HR and blood pressure, such as body weight, gender, age, and educational level. Similarly, the generalized mixed-effects model with a logit link used for dichotomized binary outcomes showed that after controlling for the effects of baseline body weight, gender, age, educational level, group, time, and the group-by-time interaction, our mHealth intervention could significantly increase the proportional rate of participants with normal SBP. In summary, these key findings demonstrate that our mHealth intervention could significantly increase medication adherence and the proportional rate of participants with normal SBP, as well as lower SBP and DBP, in patients with CHD.

In this study, participants were defined as dropping out if (1) they filled out the baseline survey but deleted the study coordinator’s WeChat account at any time point before their completion of the study; or (2) they told the study coordinator that they wanted to withdraw from the study. Participants were defined as lost to follow-up if they filled out the baseline survey and kept the study coordinator’s WeChat account, but did not fill out later surveys and did not respond to reminders and WeChat calls made by the study coordinator. This could happen at any time point during the study. In this study, 9 participants were lost to follow-up and 6 participants dropped out, which together accounts for 7% of the data. Due to the small amount of missing data, we did not perform missing data imputation. We compared the baseline variables between participants who dropped out or were lost to follow-up with those who completed the study, and found that none of the baseline variables were significantly different between these groups (Multimedia Appendix 6).

In this study, the ratio of male to female patients with CHD was 4:1, which is the same ratio as in our pilot study conducted in 2017. The higher proportion of male participants reflects the higher prevalence of CHD in men in China; Chinese men are more likely to engage in risky behaviors, such as smoking and drinking [12]. This result is consistent with findings that US men had a higher prevalence of CHD than women in all age strata from 45 to 94 years, according to the American Heart Association’s report *Heart Disease and Stroke Statistics* [50,51].

### Limitations

This study contributes important knowledge about mHealth as a tool to improve medication adherence, but it has several limitations. First, all participants were recruited at a major university-affiliated hospital; many of them were middle-aged male urban residents and were thus not representative of the general Chinese population. Second, health outcomes such as SBP, DBP, and HR were self-reported by participants. There may therefore have been discrepancies regarding these measurements. Third, during this study, some participants did not provide their health outcome data in a timely manner. To collect these data, some participants had to be reminded by WeChat messages and phone calls. These messages and phone calls might have been covariates that could influence the participants’ medication-taking behaviors, but we did not consider their influence. Finally, the mHealth intervention was not automated and lasted for only 60 days, a comparatively short period of time. To adopt this intervention in real clinical settings, which have thousands of patients, and to create long-term effects, it will be necessary to use automated methods. Finally, this study was unblinded. All participants, data collectors, and data analysts were aware of which treatment arms participants had been assigned to. To increase rigor and reduce bias, future large multisite studies should consider a double-blind design.

### Future Directions

The treatment of many chronic illnesses involves long-term pharmaceutical therapy. Nevertheless, it is an ongoing challenge to find effective ways to improve medication adherence and other health behaviors to improve health outcomes. For patients with CHD, cardioprotective medications can prevent the enlargement of harmful clots [52], cardiovascular symptoms, and poor therapeutic outcomes. Poor adherence to cardioprotective medications, however, is considered to be a public health concern [18,52]. It has been linked to increases in health care costs due to poor therapeutic outcomes that typically require major medical interventions, such as coronary angioplasty and coronary artery bypass grafting [20]. Mobile phones are widely used in China; 97% of Chinese netizens access the internet through a mobile phone [30]. This makes mobile phones an ideal platform for implementing an mHealth intervention to improve medication adherence. If this mHealth intervention could be scaled up for use in real-world clinical settings, it would have 3 useful aspects. First, it is able to overcome geographic barriers. This is particularly useful in China, considering the fact that the majority of its highly qualified health care providers reside in cities, and most of its tertiary hospitals are located in urban areas [53,54]. Hospitals in China are categorized into a 3-tier system: primary, secondary, and tertiary [55], with tertiary being the highest level of quality. To seek quality medical care, many rural residents have to travel to urban areas to find qualified health care providers. This geographic barrier limits the access of rural patients with CHD to quality medical care and may impact their health outcomes across their lifespans. Our mHealth intervention has the potential to increase the access of patients with CHD to quality medical care through mobile phones without requiring them to travel to hospitals. Second, the core technology of this mHealth intervention is app-based mobile messaging, which is widely used in China. Therefore, this intervention could be a cost-effective method to remind people across socioeconomic strata and across geographic areas to take their medications. If this intervention were automated, and if it resulted in even modest improvements in medication adherence and blood pressure, there could be population-level benefits. Given the economic burden and prevalence of CHD in China, even small clinical improvements could have a large impact. In other words, the cost-benefit ratio of this mHealth intervention is expected to be positive.

Although some studies have been conducted in China on using mobile apps (eg, WeChat) to improve blood pressure and self-management behavior [31-34], none have been designed specifically to address the problem of medication nonadherence in patients with CHD. Previous interventions have also not been individualized. For example, in an mHealth intervention study conducted in Guangzhou, educational materials were sent to a group of participants rather than to individuals [34]. Moreover, no previous study has used an mHealth intervention that integrated 2 mobile apps. Given that medication nonadherence is a complex issue involving multiple factors related to patients, health care providers, and health care systems [21], integrating mobile apps with different functionalities might be a promising approach. The mHealth intervention that we tested in this study could be harnessed and adapted to other fields, such as endocrinology, orthopedics, and mental health. Finally, adopting this intervention is integral to developing a platform for immediate medical consultation and emergency health care. Cardiovascular events such as heart attack, heart failure, arrhythmia, and heart valve problems [1] are emergencies in which many patients need immediate medical treatment, consultation, and guidance. Adopting this mHealth intervention is an important step for hospitals to develop online tools for immediate health care service.

### Conclusion

The treatment of many chronic illnesses involves long-term pharmaceutical therapy, but it is an ongoing challenge to find effective ways to improve medication adherence and promote good health outcomes. In this study, we examined an mHealth intervention to remind patients with CHD to take their cardioprotective medications. Our results demonstrate that it is feasible to conduct an mHealth intervention to improve medication adherence and health outcomes, represented by measures including SBP and DBP. In summary, mHealth interventions that are constructed using evidence-based content show promise to help increase medication adherence and improve health outcomes.

## Appendix

Multimedia Appendix 1 Comparison of changes of the proportional rates of normal SBP.

Multimedia Appendix 2 Comparison of changes of the proportional rates of normal DBP.

Multimedia Appendix 3 Frequency and proportion of normal blood pressures at multiple time points [n (%)].

Multimedia Appendix 4 Comparison of changes of the proportional rates of normal heart rate.

Multimedia Appendix 5 Results of the mixed-effects model for proportional rate change of normal health outcomes.

Multimedia Appendix 6 Baseline characteristics of participants who completed the study and those who did not [n (%)].

Multimedia Appendix 7 CONSORT-EHEALTH (V 1.6.1) checklist.

## Abbreviations

CHD: coronary heart disease
DBP: diastolic blood pressure
HR: heart rate
mHealth: mobile health
SBP: systolic blood pressure

## References

[1] (2017). What is Cardiovascular Disease?. American Heart Association.

[2] (2020). The top 10 causes of death. World Health Organization.

[3] Wilson PWF, Douglas PS (2017). Epidemiology of coronary heart disease. UpToDate.

[4] Bowry ADK, Lewey J, Dugani SB, Choudhry NK (2015). The Burden of Cardiovascular Disease in Low- and Middle-Income Countries: Epidemiology and Management. Can J Cardiol.

[5] Gaziano TA, Bitton A, Anand S, Abrahams-Gessel S, Murphy A (2010). Growing epidemic of coronary heart disease in low- and middle-income countries. Curr Probl Cardiol.

[6] (2004). The Global Burden of Disease. World Health Organization.

[7] (2018). China remains largest developing country: Economist. China Daily.

[8] China Overview. The World Bank.

[9] (2015). China: WHO statistical profile. World Health Organization.

[10] Zhang X, Lu ZL, Liu L (2008). Coronary heart disease in China. Heart.

[11] Le C, Fang Y, Linxiong W, Shulan Z, Golden AR (2015). Economic burden and cost determinants of coronary heart disease in rural southwest China: a multilevel analysis. Public Health.

[12] Chen W, Gao R, Liu L, Zhu M, Wang W, Wang Y, Wu Z, Li H, Gu D, Yang Y, Zheng Z, Jiang L, Hu S (2017). China cardiovascular diseases report 2015: a summary. J Geriatr Cardiol.

[13] Conary Heart Disease - Treatment. National Heart, Lung, and Blood Institute.

[14] (2003). Adherence to long-term therapies. World Health Organization.

[15] Hamm Christian W, Bassand Jean-Pierre, Agewall Stefan, Bax Jeroen, Boersma Eric, Bueno Hector, Caso Pio, Dudek Dariusz, Gielen Stephan, Huber Kurt, Ohman Magnus, Petrie Mark C, Sonntag Frank, Uva Miguel Sousa, Storey Robert F, Wijns William, Zahger Doron, ESC Committee for Practice Guidelines (2011). ESC Guidelines for the management of acute coronary syndromes in patients presenting without persistent ST-segment elevation: The Task Force for the management of acute coronary syndromes (ACS) in patients presenting without persistent ST-segment elevation of the European Society of Cardiology (ESC). Eur Heart J.

[16] Zhang H, Yuan X, Zhang H, Chen S, Zhao Y, Hua K, Rao C, Wang W, Sun H, Hu S, Zheng Z (2015). Efficacy of Long-Term β-Blocker Therapy for Secondary Prevention of Long-Term Outcomes After Coronary Artery Bypass Grafting Surgery. Circulation.

[17] Ho PM, Bryson CL, Rumsfeld JS (2009). Medication adherence: its importance in cardiovascular outcomes. Circulation.

[18] Jiang J, Hong T, Yu R, Zhang Y, Liu Z, Huo Y (2012). Knowledge of secondary prevention guidelines for coronary heart disease: results from a physicians' survey in China. Eur J Prev Cardiol.

[19] Ni Z, Liu C, Wu B, Yang Q, Douglas C, Shaw RJ (2018). An mHealth intervention to improve medication adherence among patients with coronary heart disease in China: Development of an intervention. International Journal of Nursing Sciences.

[20] Iuga Aurel O, McGuire Maura J (2014). Adherence and health care costs. Risk Manag Healthc Policy.

[21] Ni Z, Dardas L, Wu B, Shaw R (2019). Cardioprotective medication adherence among patients with coronary heart disease in China: a systematic review. Heart Asia.

[22] Ding R, Ma C, Chen H, Wu Y, Yang X, Hua Q, Li R, Ren W, Wang M, Xiang X, Du X, Pi L, Hu D (2013). [Control rate of increased low-density lipoprotein cholesterol levels in cardiology outpatients with coronary heart disease in Beijing]. Zhonghua Xin Xue Guan Bing Za Zhi.

[23] Zhao S, Zhao H, Wang L, Du S, Qin Y (2015). Education is critical for medication adherence in patients with coronary heart disease. Acta Cardiol.

[24] Wang H, Zhang D, Hou Z, Yan F, Hou Z (2018). Association between social health insurance and choice of hospitals among internal migrants in China: a national cross-sectional study. BMJ Open.

[25] Meng Q, Yang H, Chen W, Sun Q, Liu X (2019). People's Republic of China Health System Review.

[26] Ventola CL (2014). Mobile devices and apps for health care professionals: uses and benefits. P T.

[27] Boulos MNK, Brewer AC, Karimkhani C, Buller DB, Dellavalle RP (2014). Mobile medical and health apps: state of the art, concerns, regulatory control and certification. Online J Public Health Inform.

[28] mHealth for development: the opportunity of mobile technology for healthcare in the developing world. Vital Wave Consulting.

[29] He Y (2015). China's mobile users hit 1.3 billion in 2015.

[30] (2018). Statistical Report on Internet Development in China. China Internet Network Information Center.

[31] Dorje T, Zhao G, Scheer Anna, Tsokey Lhamo, Wang Jing, Chen Yaolin, Tso Khandro, Tan B-k, Ge Junbo, Maiorana Andrew (2018). SMARTphone and social media-based Cardiac Rehabilitation and Secondary Prevention (SMART-CR/SP) for patients with coronary heart disease in China: a randomised controlled trial protocol. BMJ Open.

[32] Dorje T, Zhao G, Tso K, Wang J, Chen Y, Tsokey L, Tan B, Scheer A, Jacques A, Li Z, Wang R, Chow CK, Ge J, Maiorana A (2019). Smartphone and social media-based cardiac rehabilitation and secondary prevention in China (SMART-CR/SP): a parallel-group, single-blind, randomised controlled trial. Lancet Digit Health.

[33] Li T, Ding W, Li X, Lin A (2019). Mobile health technology (WeChat) for the hierarchical management of community hypertension: protocol for a cluster randomized controlled trial. Patient Prefer Adherence.

[34] Li X, Li T, Chen J, Xie Y, An X, Lv Y, Lin A (2019). A WeChat-Based Self-Management Intervention for Community Middle-Aged and Elderly Adults with Hypertension in Guangzhou, China: A Cluster-Randomized Controlled Trial. Int J Environ Res Public Health.

[35] Park LG, Beatty A, Stafford Z, Whooley MA (2016). Mobile Phone Interventions for the Secondary Prevention of Cardiovascular Disease. Prog Cardiovasc Dis.

[36] Li X, Xu Z, Tang N, Ye C, Zhu X, Zhou T, Zhao Z (2016). Effect of intervention using a messaging app on compliance and duration of treatment in orthodontic patients. Clin Oral Investig.

[37] Luo L, Luo L, Zhang X, He X (2017). Hospital daily outpatient visits forecasting using a combinatorial model based on ARIMA and SES models. BMC Health Serv Res.

[38] Jacob C (1988). Statistical power analysis for the behavioural sciences (second edition).

[39] Cunningham JB, Gardner E (2007). Power, effect and sample size using GPower: practical issues for researchers and members of research ethics committees. Evidence Based Midwifery.

[40] World Health Organization 事实档案 [Facts in pictures].

[41] World Health Organization 心血管疾病 [Cardiovascular diseases].

[42] Voils CI, Maciejewski ML, Hoyle RH, Reeve BB, Gallagher P, Bryson CL, Yancy WS (2012). Initial validation of a self-report measure of the extent of and reasons for medication nonadherence. Med Care.

[43] Liu L, Writing Group of 2010 Chinese Guidelines for the Management of Hypertension (2011). [2010 Chinese guidelines for the management of hypertension]. Zhonghua Xin Xue Guan Bing Za Zhi.

[44] Schulz KF, Altman DG, Moher D, Fergusson D (2010). CONSORT 2010 changes and testing blindness in RCTs. Lancet.

[45] Harsha DW, Bray GA (2008). Weight loss and blood pressure control (Pro). Hypertension.

[46] Seimon RV, Espinoza D, Finer N, James WPT, Legler UF, Coutinho W, Sharma AM, Van Gaal L, Maggioni AP, Sweeting A, Torp-Pedersen C, Gebski V, Caterson ID (2015). Changes in body weight and pulse: outcome events in overweight and obese subjects with cardiovascular disease in the SCOUT trial. Int J Obes (Lond).

[47] Maranon R, Reckelhoff JF (2013). Sex and gender differences in control of blood pressure. Clin Sci (Lond).

[48] Reckelhoff JF (2001). Gender differences in the regulation of blood pressure. Hypertension.

[49] Buford TW (2016). Hypertension and aging. Ageing Res Rev.

[50] Mosca L, Barrett-Connor E, Wenger Nanette Kass (2011). Sex/gender differences in cardiovascular disease prevention: what a difference a decade makes. Circulation.

[51] Roger VL, Go AS, Lloyd-Jones DM, Adams RJ, Berry JD, Brown TM, Carnethon MR, Dai S, de Simone G, Ford ES, Fox CS, Fullerton HJ, Gillespie C, Greenlund KJ, Hailpern SM, Heit JA, Ho PM, Howard VJ, Kissela BM, Kittner SJ, Lackland DT, Lichtman JH, Lisabeth LD, Makuc DM, Marcus GM, Marelli A, Matchar DB, McDermott MM, Meigs JB, Moy CS, Mozaffarian D, Mussolino ME, Nichol G, Paynter NP, Rosamond WD, Sorlie PD, Stafford RS, Turan TN, Turner MB, Wong ND, Wylie-Rosett J, American Heart Association Statistics CommitteeStroke Statistics Subcommittee (2011). Heart disease and stroke statistics--2011 update: a report from the American Heart Association. Circulation.

[52] Bi Y, Gao R, Patel A, Su S, Gao W, Hu D, Huang D, Kong L, Qi W, Wu Y, Yang Y, Turnbull F, CPACS Investigators (2009). Evidence-based medication use among Chinese patients with acute coronary syndromes at the time of hospital discharge and 1 year after hospitalization: results from the Clinical Pathways for Acute Coronary Syndromes in China (CPACS) study. Am Heart J.

[53] Xie X, Liu P, Zheng Y, Zhou W, Zou J, Wang X, Wang L, Guo T, Ma X, He Y, Chen Y (2017). Equity of health resource distribution in China during 2009–15: an analysis of cross-sectional nationwide data. The Lancet.

[54] Wang H, Xu T, Xu J (2007). Factors contributing to high costs and inequality in China's health care system. JAMA.

[55] Li X, Huang J, Zhang H (2008). An analysis of hospital preparedness capacity for public health emergency in four regions of China: Beijing, Shandong, Guangxi, and Hainan. BMC Public Health.

