# Supplementary figures and images for: An mHealth Intervention to Improve Medication Adherence and Health Outcomes Among Patients With Coronary Heart Disease: Randomized Controlled Trial
Source: J Med Internet Res. 2022 Mar 9;24(3):e27202. doi: 10.2196/27202 (PMC8943565; doi:10.2196/27202)

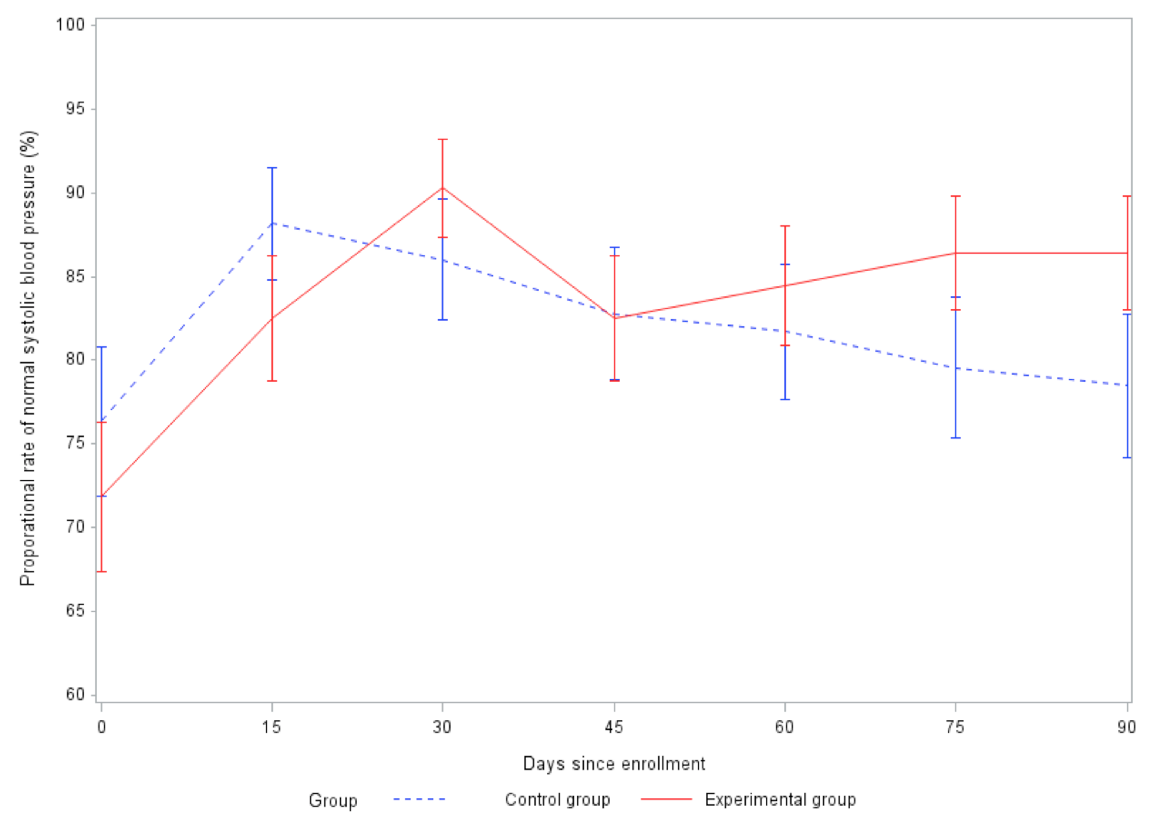

Supplement: Multimedia Appendix 1 [file jmir_v24i3e27202_app1.png]

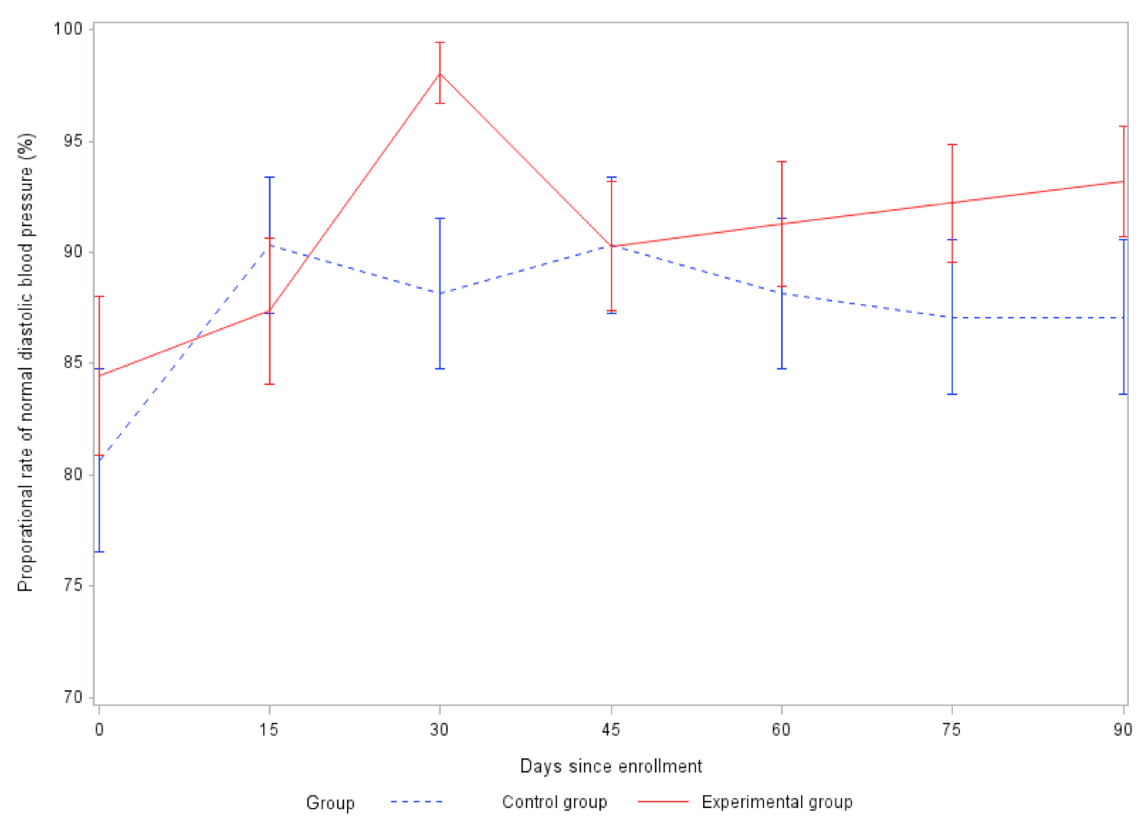

Supplement: Multimedia Appendix 2 [file jmir_v24i3e27202_app2.png]

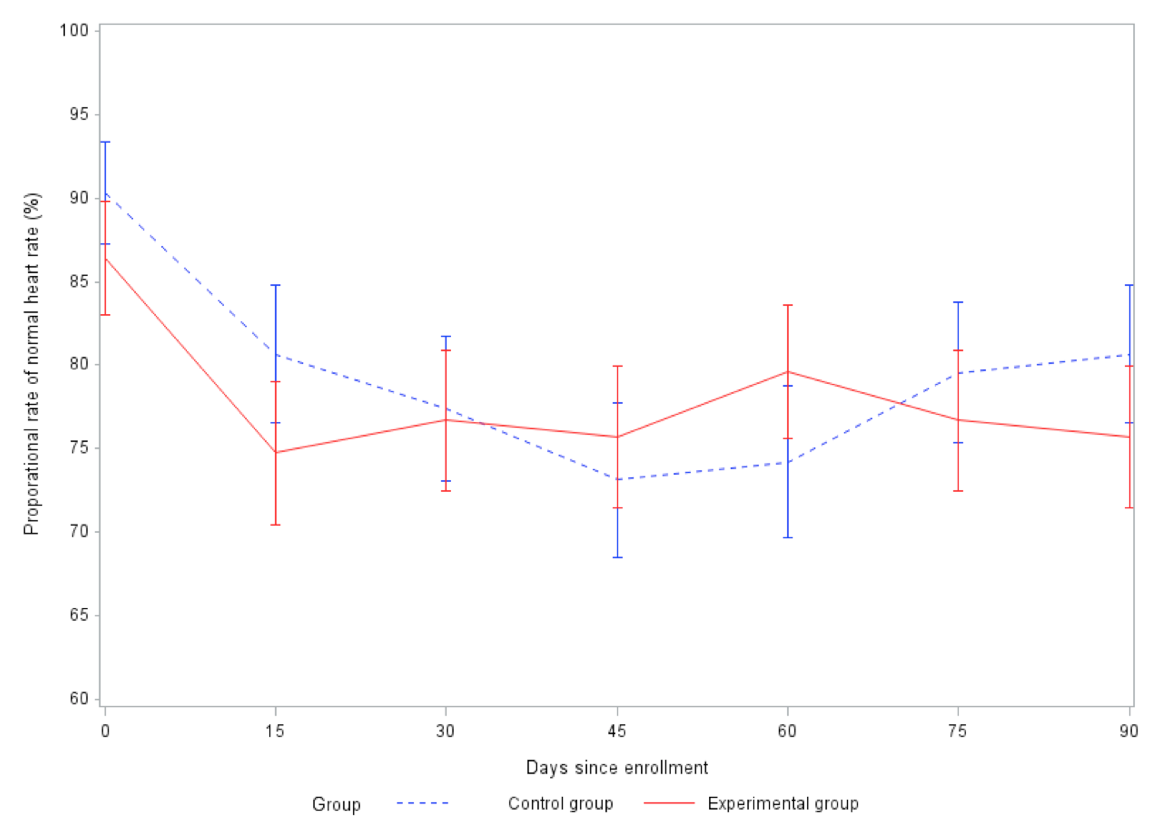

Supplement: Multimedia Appendix 4 [file jmir_v24i3e27202_app4.png]
