# Supplementary material for: An mHealth Intervention to Improve Medication Adherence and Health Outcomes Among Patients With Coronary Heart Disease: Randomized Controlled Trial
Source: J Med Internet Res. 2022 Mar 9;24(3):e27202. doi: 10.2196/27202 (PMC8943565; doi:10.2196/27202)
Supplement: Multimedia Appendix 3 [file jmir_v24i3e27202_app3.pdf]

	All participants (*N*=196)	Experimental (*n*=103)	Control (*n*=93)	*P* value
**Systolic blood pressure (mmHg)**				
baseline	145 (73.98)	74 (71.84)	71 (76.34)	.47
60 days	163 (83.16)	87 (84.47)	76 (81.72)	.61
90 days	162 (82.65)	89 (86.41)	73 (78.49)	.14
**Diastolic blood pressure (mmHg)**				
baseline	162 (82.65)	87 (84.47)	75 (80.65)	.48
60 days	176 (89.80)	94 (91.26)	82 (88.17)	.48
90 days	179 (91.33)	96 (93.20)	81 (87.10)	.15
**Heart rate (bpm)**				
baseline	173 (88.27)	89 (86.41)	84 (90.32)	.40
60 days	145 (73.98)	82 (79.61)	69 (74.19)	.37
90 days	141 (71.94)	78 (75.73)	75 (80.65)	.41
