# Supplementary material for: An mHealth Intervention to Improve Medication Adherence and Health Outcomes Among Patients With Coronary Heart Disease: Randomized Controlled Trial
Source: J Med Internet Res. 2022 Mar 9;24(3):e27202. doi: 10.2196/27202 (PMC8943565; doi:10.2196/27202)
Supplement: Multimedia Appendix 5 [file jmir_v24i3e27202_app5.pdf]

Item	Diastolic blood pressure		Systolic blood pressure		Heart rate	
	**Parameter estimation**	***P* value**	**Parameter estimation**	***P* value**	**Parameter estimation**	***P* value**
Intercept	0.43	.83	7.06	<.001	-1.13	.61
Group (reference=control)	0.15	.71	-0.55	.17	-0.16	.73
Gender (reference=male)	0.32	.52	0.03	.94	-0.53	.27
Age	0.04	.003	-0.04	.007	0.05	.003
Education	-0.03	.77	-0.11	.37	0.20	.13
Weight	-0.02	.20	-0.03	.05	0.01	.47
Time	0.03	.01	0.03	.01	-0.04	<.001
Time*time	-0.0003	.01	-0.0003	.003	0.0004	<.001
Group*time	0.006	.32	0.01	.02	0.003	.60
