# Supplementary material for: An mHealth Intervention to Improve Medication Adherence and Health Outcomes Among Patients With Coronary Heart Disease: Randomized Controlled Trial
Source: J Med Internet Res. 2022 Mar 9;24(3):e27202. doi: 10.2196/27202 (PMC8943565; doi:10.2196/27202)
Supplement: Multimedia Appendix 6 [file jmir_v24i3e27202_app6.pdf]

Variable	All participants	Completed	Dropped out or lost to follow-up	*P* value
Number of participants	196 (100)	181 (92.3)	15 (7.7)	
**Gender**				.74
Male	157 (80.1)	144 (79.6)	13 (86.7)	
Female	39 (19.9)	37 (20.4)	2 (13.3)	
**Weight (kg),** *Mean ± SD*	67.6 ± 11.3	67.7 ± 11.3	66.6 ± 10.4	.67
**Height (cm),** *Mean ± SD*	164.8 ± 8.0	164.7 ± 8.1	166.7 ± 7.2	.48
**Age (year),** *Mean ± SD*	61 ± 11	61 ± 11	59 ± 13	.46
**Ethnicity**				.05
Han	184 (93.9)	172 (95.0)	12 (80.0)	
Other ethnic minorities	12 (6.1)	9 (5.0)	3 (20.0)	
**Marital status**				.32
Married	181 (92.4)	168 (92.8)	13 (86.7)	
Widowed, separated, divorced, or single	15 (7.6)	13 (7.2)	2 (7.2)	
**Job status**				.69
Employed	65 (34.0)	60 (33.7)	5 (38.5)	
Unemployed	4 (2.1)	4 (2.3)	0	
Farmer	19 (10.0)	19 (10.7)	0	
Retired	103 (53.9)	95 (53.4)	8 (61.5)	
**Education**				.97
Primary school or lower	31 (16.1)	29 (16.2)	2 (14.3)	
Middle school	43 (22.3)	40 (22.4)	3 (21.4)	
High school	47 (24.3)	43 (24.0)	4 (28.6)	
High school—College	28 (14.5)	27 (15.1)	1 (7.1)	
College or above	44 (22.8)	40 (22.4)	4 (28.6)	
**Number of prescribed medications**				1.00
< 5	75 (38.3)	69 (38.1)	6 (40.0)	
5 ≤ < 10	116 (59.2)	107 (59.1)	9 (60.0)	
≥ 10	5 (2.5)	5 (2.8)	0	
**Have medical insurance**	186 (96.9)	172 (95.0)	14 (93.3)	1.00
**Medications were covered by medical insurance**				.75
All covered	41 (27.9)	38 (27.5)	3 (33.3)	
Some covered	89 (60.5)	84 (60.9)	5 (55.6)	
Not covered at all	17 (11.6)	16 (11.6)	1 (11.1)	
**Place of residence**				.73
Urban	161 (82.1)	149 (82.3)	12 (80.0)	
Rural	35 (17.9)	32 (17.7)	3 (20.0)	
**Living arrangement**				1.00
Living alone	14 (7.1)	13 (7.2)	1 (6.7)	
With family or relatives	182 (92.9)	168 (92.8)	14 (93.3)	

**Length of using WeChat (in years)**				.80
< 1	24 (12.0)	23 (12.7)	1 (6.7)	
1 ≤ < 5	109 (56.0)	101 (55.8)	8 (53.3)	
≥ 5	63 (32.0)	57 (31.5)	6 (40.0)	
**Frequency of WeChat use before participating in the study**				.76
Daily	152 (80.9)	139 (80.4)	13 (86.7)	
Occasionally	25 (13.3)	24 (13.9)	1 (6.7)	
Never	11 (5.8)	10 (5.8)	1 (6.7)	
**Yearly family income (Chinese Yuan)**				.78
< 54,000	81 (52.3)	74 (52.9)	7 (46.7)	
54,001 – 90,000	34 (21.9)	29 (20.7)	5 (33.3)	
90,001 – 120,000	18 (11.6)	18 (12.9)	0	
> 120,000	22 (14.2)	19 (13.6)	3 (20.0)	
**General health status**				.92
Good	70 (35.7)	64 (35.4)	6 (40.0)	
Fair	101 (51.5)	94 (51.9)	7 (46.7)	
Bad	25 (12.8)	23 (12.7)	2 (13.3)	
